# Frequencies of BCR-ABL1 fusion transcripts among Sudanese chronic myeloid leukaemia patients

**DOI:** 10.1590/S1415-47572010005000037

**Published:** 2010-06-01

**Authors:** Emad-Aldin I. Osman, Kamal Hamad, Imad M. Fadl Elmula, Muntaser E. Ibrahim

**Affiliations:** 1 Department of Molecular Biology, Institute of Endemic Diseases, University of Khartoum, KhartoumSudan; 2 Faculty of Medicine, University of Khartoum, KhartoumSudan; 3 Faculty of Medical Laboratories sciences, Al-Neelain University, KhartoumSudan

**Keywords:** BCR-ABL, chronic myeloid leukemia, Ph chromosome, Sudanese, RT-PCR

## Abstract

The incidence of one or other rearrangement in chronic myeloid leukemia (CML) patients varies in different reported series. In this study we report the frequencies of BCR*-*ABL1 fusion transcript variants studied in 43 CML patients from Sudan. The study includes 46 Sudanese patients, three of which negative for the BCR-ABL1 fusion transcript. More than half of 43 positive patients showed b2a2 fusion transcript (53.5%), while (41.9%) showed b3a2 transcript and the remaining (4.6%) coexpression of b3a2/ b2a2 and b3a2/b2a2/e19a2. We detected neither coexpression of p210/p190 nor e1a2 alone. Male patients showed a tendency to express b2a2, while female tende to express b3a2 (p = 0.017). Moreover, a single nucleotide polymorphism was detected in BCR exon 13 in one out of four patients and this patient showed only b2a2 expression. In conclusion, we observed a significant correlation between sex and type of BCR-ABL1 transcript, an observation that deserves further investigation.

The Philadelphia (Ph) chromosome was the first chromosomal abnormality to be associated with a specific malignant disease in humans, namely chronic myeloid leukemia (CML). This is a shortened chromosome 22 resulting from reciprocal translocation, t(9;22)(q34;q11), between the long arms of chromosomes 9 and 22. This translocation results in the formation of two hybrid genes, BCR-ABL1 on the Ph chromosome and ABL1-BCR on 9q+ (Melo, 1996). A Ph chromosome is the hallmark of CML and is found in up to 95% of the patients. It is also found in 5% of children and in 15 to 30% of adults with acute lymphoid leukemia, and in 2% of patients with newly diagnosed acute myeloblastic leukemia (Kurzrock *et al.*, 1988).

In most CML patients, the break in chromosome 22 is restricted to an area of 5.8-kb termed M-bcr. M-bcr consists of five exons termed M-bcr exons b1-b5. These exons are actually located within the central region of the BCR gene and are equivalent to exons 12-16 (e12- e16) of this gene. Most breaks occur immediately downstream of exon 2 or 3 of the M-bcr region and result in b2a2 or b3a2 fusion transcripts (Groffen *et al.*, 1984). A very small proportion of Ph-positive CML patients display a larger BCR-ABL1 fusion transcript that results from a fusion between BCR exon 19 (originally named c3) and ABL exon 2. This is caused by breakpoints in the μ-breakpoint cluster region (μ-bcr) between BCR exons 19 and 20 (van Dongen *et al.*, 1999). This e19a2 (c3/a2) junction yields a transcript that contains an additional BCR sequence of 540 bp and encodes for a chimeric protein carrying 180 additional amino acids, as compared with that found in typical CML (Pane *et al.*, 1996).

In 60 to 80% of all patients with Ph-positive ALL, the breakpoint occurs in the first intron of the BCR gene, in a region referred to as the minor breakpoint cluster region (m-bcr), thereby producing the shorter isotype p190 BCR-ABL1 from the e1a2 type mRNA (Deininger *et al.*, 2000). Although the e1a2 type of transcript has been mainly associated with ALL, sporadic cases of CML expressing only this type of transcript have also been reported (Selleri *et al.*, 1990).

The majority of CML patients have transcripts with the b3a2 (55%) or b2a2 (40%) junctions. In 5% of the cases, both b3a2 and b2a2 transcripts can be formed as a result of alternative splicing (Melo, 1996).

This study was carried out to determine the frequency of expression of BCR-ABL1 fusion transcript variants in Sudanese CML patients by using RT-PCR, and was partly motivated by the introduction of imatinib mesylate, whose administration is currently based on the molecular diagnosis of BCR-ABL1 fusion genes.

Blood samples from 46 patients with preliminary diagnosis of CML for the period 2004 to 2008, were obtained for confirmation by molecular studies. Preliminary diagnosis was carried out according to clinical presentation and morphological criteria of blood and bone marrow. Written informed consent was obtained from all the patients or family members.

Venous blood (5 mL) in EDTA tubes was collected from the CML patients. Buffy coat was isolated and washed in red cells lysis buffer. Total RNA was extracted from about 10^6^ white cells by Trizol (Invitrogen). RNA integrity was determined by gel electrophoresis prior to reverse transcription.

For cDNA synthesis, the concentration of RNA was first measured spectrophotometrically, and then the cDNA was synthesized using M-MuLV Reverse Transcriptase and other reaction components (Fermentas). 2 μg of RNA were reverse transcribed with 200 units of M-MuLV RT in a reaction mix consisting of 1x RT buffer (50 mM Tris-HCl , 50 mM KCl, 4 mM MgCl_2_, 10 mM DTT), 0.5 μg of oligo(dT)_18_, 20 mM dNTP and 20 units RNase inhibitor, in a final volume of 20 μL. Reaction conditions were 70 °C for 5 min, 37 °C for 5 min, 42 °C for 1 h and 70 °C for 10 min.

BCR-ABL1 transcripts were detected by PCR, using allele-specific primers for p210 and p190 primer sequences, as already described (van Dongen *et al.*, 1999). The (A2) primer ‘5 TTC AGC GGC CAG TAG CAT CTG ACT T 3' was also used for ABL1 as forward together with a reverse primer. PCR was carried out in a total volume of 23 μL of a reaction mixture containing 2.5 μL of 10X PCR buffer (10 mM Tris-HCl pH 8.3, 50 mM KCl), 2.5 μL of 25 mM MgCl_2_, 1 μL of 10 mM dNTPs, 2 μL of 10 μM of forward and reverse primers, 14 μL of H_2_O and 1 μL of 1U/μL *Taq* DNA polymerase. A touchdown program was employed, under the following conditions: an initial denaturation step at 95 °C for 3 min., then followed by 14 cycles of denaturing at 94 °C for 20 s, primer annealing at 64 °C and a decrease 0.5 °C per cycle for 30 s, extension at 72 °C for 45 s, then 35 cycle as following: denaturing at 94 °C for 20 s, primer annealing at 58 °C for 30 s, extension at 72 °C for 45 s and a final extension step at 72 °C for 5 min. PCR was done using separate reactions for each pair of primers. The PCR products were visualized directly on ethidium bromide-stained 2% agarose gel. ABL1 gene was used as internal control. Bands of 417 bp, 342 bp, 957 bp and 226 bp were observed for b3a2, b2a2, e19a2 and normal ABL1, respectively.

The PCR products were sent for commercial sequencing at Macrogen Company (South Korea).

The Chi square method was applied for comparing obtained BCR-ABL1 variants of the CML patients by gender.

Of the 46 patients studied, 43 were positive for one or more of the BCR-ABL1 rearrangements. The b2a2 transcript was detected in 23 (53.5%) of the CML patients, b3a2 in 18 (41.9%), both b2a2 and b3a2 in one (2.3%) patient, and b2a2, b3a2 with e19a2 also in one (2.3%) patient. The e1a2 transcript was not detected. Of the 43 CML patients, 20 were males, 15 expressing b2a2, and 23 females, 13 of which expressing b3a2, and two females showed co-expression. We found a correlation between the obtained BCR-ABL1 variants and the sex type of the CML patients (p = 0.017). Table 1 shows the BCR-ABL1 transcript types and the patients' gender.

The distribution of transcript type in CML has been studied in European and some other populations (Eisenberg *et al.*, 1988; Lee *et al.*, 1989) with frequencies for b2a2 and b3a2 transcripts being roughly of the order of 40% and 55%, and that for co-expression of b3a2 and b2a2 representing 5% of the cases. A study on an Ecuadorian population, however, registered very different frequencies: 5% for b3a2 and 95% for b2a2 (Paz-y-Mino *et al.*, 2002). In the present study, a frequency of 53.5% and 41.9% for b2a2 and b3a2, respectively, was established, values which are is relatively closer to those from a Mexican population (Arana-Trejo *et al.*, 2002). This difference in frequencies may be due to the genetic background of the populations.

Furthermore, it was found that CML patients at diagnosis also expressed either low e1a2 transcript frequency, besides the usual BCR-ABL1 p210 (Saglio *et al.*, 1996; Van Rhee *et al.*, 1996), or only 5% (Arana-Trejo *et al.*, 2002), or no co-expression whatsoever (Yaghmaie *et al.*, 2008). In this study we did not detect co-expression of p210 and p190 in any of the Sudanese patients. This may be due to technique sensitivity, although ethnic differences cannot be disregarded.

We found a significant correlation between sex and transcript type, in which male patients showed a higher tendency of expressing b2a2 and females a higher tendency for b3a2 (p = 0.017). This is in agreement with a study by Adler *et al.* (2009), which included 146 paediatric patients with CML and reported the following proportions for the b2a2 transcript [34 males (51%) *vs.* 21 females (27%)], and inversely for the b3a2 [17 males (25%) *vs.* 36 females (45%)]. In all these cases, the sex-dependent skewed distribution in BCR-ABL1 transcript types deserves further investigation.

In this study, only two patients (4.6%) manifested co-expression, one expressed b3a2/b2a2 and the other b3a2/b2a2 with e19a2. Co-expression may arise either from alternative splicing, or the existence of several leukemia cell lines with different BCR-ABL1 transcript expression.

Co-expression of b2a2 and b3a2 transcripts has been linked to two polymorphisms, T to C at exon 13 and A to G at intron 13 (Meissner *et al.*, 1998; Branford *et al.*, 2002). Six PCR products from four patients were sequenced to confirm the products of four b2a2 and two b3a2 and one was found to harbor T to C at exon 13 and expressed only b2a2 transcript which might indicate that this exonic polymorphism is not obligatory for co-expression, as reported by Mondal *et al.* (2006). Moreover, this polymorphism has no implication on the primary structure of BCR and BCR-ABL1 proteins. However, since the alteration is located close to the fusion region, it may have a significant influence on the annealing of PCR primers, probes for real time PCR, and antisense oligonucleotides. We intend to study this polymorphism in a larger set of CML samples.

Finally, the difference in size of BCR-ABL1 transcripts is not only a reflection of variation at the sites of breakage/fusion, but is also a result of alternative splicing between BCR and ABL and within BCR itself.

## Figures and Tables

**Table 1:** Incidence of BCR-ABL1 transcripts and gender.

BCR-ABL re-arrangement	Case (%)	Gender (male/female)
b2a2	23 (53.5%)	15/8*
b3a2	18 (41.9%)	5/13*
b2a2/b3a2	1 (2.3%)	0/1
b2a2/b3a2/e19a2	1 (2.3%)	0/1

*p = 0.017.

## References

[Adleretal2009] Adler R., Viehmann S., Kuhlisch E., Martiniak Y., Rottgers S., Harbott J., Suttorp M. (2009). Correlation of BCR/ABL transcript variants with patients' characteristics in childhood chronic myeloid leukaemia. Eur J Haematol.

[Arana-Trejoetal2002] Arana-Trejo R.M., Ruiz Sanchez E., Ignacio-Ibarra G., Baez de la Fuente E., Garces O., Gomez Morales E., Castro Granados M., Ovilla Martinez R., Rubio-Borja M.E., Solis Anaya L. (2002). BCR/ABL p210, p190 and p230 fusion genes in 250 Mexican patients with chronic myeloid leukaemia. Clin Lab Haematol.

[Branfordetal2002] Branford S., Hughes T.P., Rudzki Z. (2002). Dual transcription of b2a2 and b3a2 BCR-ABL transcripts in chronic myeloid leukaemia is confined to patients with a linked polymorphism within the BCR gene. Br J Haematol.

[Deiningeretal2000] Deininger M.W., Goldman J.M., Melo J.V. (2000). The molecular biology of chronic myeloid leukemia. Blood.

[Eisenbergetal1988] Eisenberg A., Silver R., Soper L., Arlin Z., Coleman M., Bernhardt B., Benn P. (1988). The location of breakpoints within the breakpoint cluster region of chromosome 22 in chronic myeloid leukemia. Leukemia.

[Groffenetal1984] Groffen J., Stephenson J.R., Heisterkamp N., de Klein A., Bartram C.R., Grosveld G. (1984). Philadelphia chromosomal breakpoints are clustered within a limited region, bcr, on chromosome 22. Cell.

[Kurzrocketal1988] Kurzrock R., Gutterman J.U., Talpaz M. (1988). The molecular genetics of Philadelphia chromosome-positive leukemias. N Engl J Med.

[Leeetal1989] Lee M.S., LeMaistre A., Kantarjian H.M., Talpaz M., Freireich E.J., Trujillo J.M., Stass S.A. (1989). Detection of two alternative bcr/abl mRNA junctions and minimal residual disease in Philadelphia chromosome positive chronic myelogenous leukemia by polymerase chain reaction. Blood.

[Meissneretal1998] Meissner R.V., Dias P.M., Covas D.T., Job F., Leite M., Nardi N.B. (1998). A polymorphism in exon b2 of the major breakpoint cluster region identified in chronic myeloid leukaemia patients. Br J Haematol.

[Melo1996] Melo J.V. (1996). The diversity of BCR-ABL fusion proteins and their relationship to leukemia phenotype. Blood.

[Mondaletal2006] Mondal B.C., Bandyopadhyay A., Majumdar S., Mukhopadhyay A., Chandra S., Chaudhuri U., Chakrabarti P., Bhattacharyya S., Dasgupta U.B. (2006). Molecular profiling of chronic myeloid leukemia in Eastern India. Am J Hematol.

[Paneetal1996] Pane F., Frigeri F., Sindona M., Luciano L., Ferrara F., Cimino R., Meloni G., Saglio G., Salvatore F., Rotoli B. (1996). Neutrophilic chronic myeloid leukemia: A distinct disease with a specific molecular marker (BCR/ABL with C3/A2 junction). Blood.

[Paz-y-Minoetal2002] Paz-y-Mino C., Burgo R., Morillo S.A., Santos J.C., Fiallo B.F., Leone P.E. (2002). BCR-ABL rearrangement frequencies in chronic myeloid leukemia and acute lymphoblastic leukemia in Ecuador, South America. Cancer Genet Cytogenet.

[Saglioetal1996] Saglio G., Pane F., Gottardi E., Frigeri F., Buonaiuto M.R., Guerrasio A., De Micheli D., Parziale A., Fornaci M.N., Martinelli G. (1996). Consistent amounts of acute leukemia-associated P190 bcr/abl transcripts are expressed by chronic myelogenous leukemia patients at diagnosis. Blood.

[Sellerietal1990] Selleri L., von Linderen M., Hermans A., Meijer D., Torelli G., Grosveld G. (1990). Chronic myeloid leukemia may be associated with several bcr/abl transcripts including the acute lymphoid leukemia type 7 kb transcript. Blood.

[vanDongenetal1999] van Dongen J.J., Macintyre E.A., Gabert J.A., Saglio G., Gottardi E., Rambaldi A., Dotti G., Griesinger F., Parreira A., Gameiro P. (1999). Standardized RT-PCR analysis of fusion gene transcripts from chromosome aberrations in acute leukemia for detection of minimal residual disease. Report of the BIOMED-1 concerted action: Investigation of minimal residual disease in acute leukemia. Leukemia.

[VanRheeetal1996] Van Rhee F., Hochhaus A., Lin F., Melo J.V., Goldman J.M., Cross N.C. (1996). p190 BCR/ABL mRNA is expressed at low levels in p210- positive chronic myeloid and acute lymphoblastic leukemias. Blood.

[Yaghmaieetal2008] Yaghmaie M., Ghaffari S.H., Ghavamzadeh A., Alimoghaddam K., Jahani M., Mousavi S.A., Irvani M., Bahar B., Bibordi I. (2008). Frequency of BCR-ABL Fusion transcripts in Iranian patients with Chronic Myeloid Leukemia. Arch Iran Med.

